# Response to Proton‐Pump Inhibitors Therapy in Pediatric Patients With Eosinophilic Esophagitis in Bogotá, Colombia

**DOI:** 10.1155/cjgh/4633813

**Published:** 2025-11-25

**Authors:** Andrea Estefanía Rodríguez López, José Fernando Vera Chamorro, Ailim Margarita Carias Domínguez, Mónica Viviana Pinilla Roncancio, Rocío del Pilar López Panqueva, Gonzalo Andrés Montaño Rozo

**Affiliations:** ^1^ School of Medicine, Universidad de Los Andes, Bogotá, Colombia, uandes.cl; ^2^ Department of Pediatrics, Fundación Santa Fe de Bogotá, Bogotá, Colombia, fsfb.org.co; ^3^ Department of Anatomic Pathology and Clinical Laboratory, Fundación Santa Fe de Bogotá, Bogotá, Colombia, fsfb.org.co

## Abstract

**Introduction:**

Eosinophilic esophagitis (EoE) is a chronic immune‐mediated disease and a leading cause of food impaction in pediatric populations, with potential functional and structural complications. Proton‐pump inhibitors (PPIs) are widely used as first‐line therapy, but the response rate in Colombian children remains unknown. This study aimed to determine the initial histologic response to PPI therapy and describe clinical, endoscopic, and histological characteristics in a sample of pediatric patients with EoE from Bogotá, Colombia.

**Methodology:**

A retrospective observational longitudinal study of patients aged 2–18 years diagnosed with EoE at a tertiary care center between 2015 and 2022 was conducted. Patients underwent initial clinical and endoscopic assessment confirming esophageal eosinophilia (≥ 15 eosinophils per high‐power field [eos/hpf]), followed by at least 8‐week course of PPI therapy and a subsequent clinical‐endoscopic re‐evaluation. Treatment response was defined histologically as < 15 eos/hpf in follow‐up esophageal biopsy. Demographic, clinical, endoscopic, and histological data were analyzed using descriptive and bivariate statistics.

**Results:**

We included 34 patients (median age 11 years; 61.8% boys). Sixteen (47%) achieved histological remission with initial PPI therapy. Being esomeprazole preferred in 88%, with median dose 1.37 mg/kg/day. No significant differences were observed between responders and nonresponders in demographic variables, familial and personal atopic history, symptoms, or endoscopic findings. Histologically, responders demonstrated significant reductions in eosinophil counts, as well as improvements in basal zone hyperplasia, eosinophilic abscesses, and eosinophil surface layering (all *p* < 0.005).

**Discussion:**

This is the first study to assess PPI response in Colombian pediatric EoE patients, demonstrating a response rate consistent with existing literature. Histologic evaluation was identified as the most reliable marker of treatment success, underscoring the critical role of histopathological follow‐up in this disease.

## 1. Introduction

Eosinophilic esophagitis (EoE) is a chronic immune‐mediated disease, characterized clinically by age‐dependent manifestations of esophageal dysfunction and histologically by the presence of more than 15 eosinophils per high‐power field (eos/hpf) in esophageal biopsies [1, 2].

Over recent decades, its incidence and prevalence have increased, making it the most common cause of chronic esophagitis in children and the leading etiology of dysphagia and food impaction in children and adolescents [1–3]. Prevalence in pediatrics is estimated between 0.5 and 1 per 1000 children [4]. In Latin America, estimates reach 3.69 cases per 1000 children, with Colombia reporting one of the highest rates in the region, with 18.2 cases per 1000 children [5].

Management strategies for EoE have evolved considerably, particularly regarding proton‐pump inhibitors (PPIs) in the diagnosis and control of the disease. Initially, PPIs were used as a diagnostic tool to distinguish gastroesophageal reflux disease (GERD) as an independent entity [2, 6]. More recently, PPIs have been recognized as a first‐line treatment along with dietary restriction regimens and topical esophageal steroids [2, 7, 8]. PPIs are now widely used first‐line option in clinical practice [8], with reported histological remission rates ranging from 23% to 83% with large studies reporting rates around 50% in pediatric population [7, 8].

Despite growing epidemiological data, there is a lack of studies evaluating histological response to PPI therapy in pediatric EoE in Latin America, and no such data have been published from Colombia. Therefore, this study aims to analyze the response to initial PPI treatment and describe the clinical, endoscopic, and histological characteristics related to this response in a sample of pediatric patients with EoE in a referral pediatric gastroenterology center in Bogotá, Colombia.

## 2. Methodology

### 2.1. Study Design

This is a retrospective, longitudinal, observational study.

### 2.2. Participants

Pediatric patients aged 2–18 years diagnosed with EoE who attended the Pediatric Gastroenterology Division at Fundación Santa Fe de Bogotá (FSFB) in Colombia between January 2015 and December 2022 were eligible for inclusion.

Inclusion criteria required patients to have undergone two clinical and endoscopic follow‐ups: patients initially presenting with symptoms of esophageal dysfunction, confirmed by an esophagogastroduodenoscopy (EGD) revealing esophageal eosinophilia (≥ 15 eos/hpf) in either proximal or distal esophageal biopsies. Subsequently, patients received a PPI regimen, including esomeprazole, omeprazole or pantoprazole at dosages between 1 and 2 mg/kg/day for a minimum of 8 weeks, followed by a second clinical and endoscopic follow‐up during PPI therapy.

Exclusion criteria encompassed failure to complete both follow‐ups, documented nonadherence to treatment, allergies or contraindications to PPI, use of other therapeutic modalities (elimination diets or topical steroids), incomplete clinical records or lack of institutional follow‐up, and secondary esophageal eosinophilia attributable to other systemic diseases (e.g., Crohn’s disease, hypereosinophilic syndrome, or eosinophilic colitis/gastroenteritis).

### 2.3. Sample

A consecutive nonprobabilistic sampling approach was applied, drawing from institutional pediatric gastroenterology and pathology databases at FSFB from 2015 to 2022 period.

### 2.4. Data and Variables

Comprehensive review of medical records, endoscopy, and histopathology reports was conducted to extract the following variables:

• Demographics: sex and age (with stratified age over and under 10 years, based on mean age).
• Personal and parental atopic history (including asthma, allergic rhinitis, atopic dermatitis, self‐reported food allergies, and self‐reported aeroallergens sensitization).
• Relevant comorbidities such as gastroesophageal reflux, gastritis, malnutrition, prematurity, and mode of delivery.
• Clinical manifestations of esophageal dysfunction at baseline and after 8 weeks of PPI therapy, as reported by patients or parents (for younger children), following consensus diagnostic criteria [2].
• Endoscopic findings using the EoE endoscopic reference score (EREFS) at baseline and posttreatment [9, 10].
• Histological parameters including eosinophil count in proximal and distal esophageal specimens and presence/absence of other histological features based on the eosinophilic esophagitis histology scoring system (EoEHSS) at baseline and after PPI treatment [11].

The primary outcome was histological response to PPI therapy, defined as < 15 eos/hpf in all esophageal biopsies at follow‐up. Nonresponders were those with persistent eosinophilia (≥ 15 eos/hpf) in any biopsy site.

### 2.5. Statistical Analysis

Categorical variables are presented as frequencies and percentages, and continuous variables as medians and interquartile ranges (IQRs). Comparisons between responders and nonresponders used the Mann–Whitney *U* test for continuous variables and Fisher’s exact test for categorical data. Statistical significance was set at *p* < 0.05.

Data were managed in REDCAP® software and statistical analysis was performed in STATA statistical software Version 17.0® (StataCorp, College Station, TX USA), licensed by Los Andes University.

### 2.6. Ethical Aspects

Given its retrospective nature, the study was classified a minimal risk investigation. The protocol was approved by the FSFB Medical Ethics Committee. Reporting follows STROBE guidelines for observational studies.

## 3. Results

**Figure 1:**
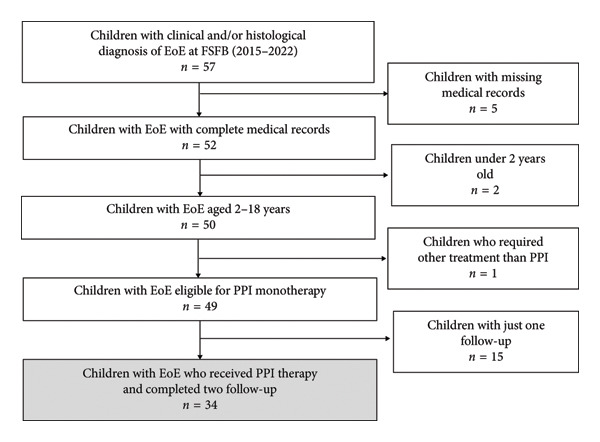
Selection flowchart.

A total of 34 participants met the inclusion criteria (Figure 1), with a predominance of boys (61.8%) and a median age of 11 years (IQR: 8–14) (Table 1). All participants received PPI therapy at a median dose of 1.37 mg/kg/day with esomeprazole administered twice daily as the preferred regimen in 88% of the cases.

**Table 1:** Demographic and medical history of children included in the study.

	General % (*n*) *n* = 34	PPI treatment response % (*n*)		
		Responders *n* = 16	Nonresponders *n* = 18	*p* value
Sex				
(i) Boys	61.76% (21)	50% (8)	72.22% (13)	0.291^**a**^
(ii) Girls	38.24% (13)	50% (8)	27.78% (5)	
Median age (IQR)	11 (8–14)	10.56 (8.42–12.7)	10.77 (8.98–12.5)	0.938^**b**^
Age group				
(i) School children (< 10 years)	41.18% (14)	43.75% (7)	38.89% (7)	0.524^**a**^
(ii) Teenagers (> 10 years)	58.82% (20)	56.25% (9)	61.11% (11)	
Parents atopic history				
(i) Eosinophilic esophagitis	2.94% (1)	6.25% (1)	0	0.471^**a**^
(ii) Asthma	23.53% (8)	12.5% (2)	33.33% (6)	0.153^**a**^
(iii) Allergic rhinitis	47.06% (16)	50% (8)	44.44% (8)	0.508^**a**^
(iv) Atopic dermatitis	17.65% (6)	18.75% (3)	16.67% (3)	0.611^**a**^
(v) Food allergies	11.76% (4)	0	22.22% (4)	0.066^a^
Personal medical history				
(i) Asthma	73.53%(25)	68.75% (11)	77.78% (14)	0.417^**a**^
(ii) Allergic rhinitis	76.47% (26)	68.75% (11)	83.33% (15)	0.276^**a**^
(iii) Atopic dermatitis	38.24% (13)	31.25% (5)	44.44% (8)	0.332^**a**^
(iv) Food allergies	70.59% (24)	62.5% (10)	77.78% (14)	0.275^**a**^
(1) Cow’s milk protein allergy	52.9% (18)	43.75% (7)	61.11% (11)	0.494^**a**^
(2) Egg	29.41% (10)	37.5% (6)	22.22% (4)	0.132^**a**^
(3) Wheat	5.88% (2)	6.25% (1)	5.56% (1)	0.670^**a**^
(4) Soy	8.82% (3)	12.5% (2)	5.56% (1)	0.371^**a**^
(5) Fish/seafood	8.82% (3)	12.5% (2)	5.56% (1)	0.371^**a**^
(6) Peanuts/nuts	20.59% (7)	18.75% (3)	22.22% (4)	0.643^**a**^
(v) Sensitivity to aeroallergens	20.59% (7)	18.75% (3)	22.22% (4)	0.571^**a**^
(vi) Gastroesophageal reflux	20.59% (7)	6.25% (1)	33.33% (6)	0.061^**a**^
(vii) Gastritis	52.94% (18)	56.25% (9)	50% (9)	0.492^**a**^
(viii) Low weight/malnutrition	17.65% (6)	18.75% (3)	16.67% (3)	0.611^**a**^
(ix) Prematurity	8.82% (3)	6.25% (1)	11.11% (2)	0.545^**a**^
(x) Cesarean birth	38.24% (13)	37.5% (6)	38.89% (7)	0.607^a^

*Note:* Statistical significance at 5%.

Abbreviation: IQR = interquartile range.

^a^Fisher’s exact test.

^b^Mann–Whitney *U* test.

### 3.1. Atopic History and Comorbidities

Allergic rhinitis was the most frequently reported atopic condition among parents, while among patients, allergic rhinitis, asthma, and food allergies were most common. Cow’s milk protein allergy (CMPA) and egg allergy were the most prevalent food allergies. Among gastrointestinal comorbidities gastritis was most prevalent, followed by gastroesophageal reflux (Table 1).

### 3.2. Clinical Presentation

At baseline, heartburn, abdominal pain, and regurgitation were the most common symptoms (Table 2). When symptoms were stratified by age, weight loss or poor weight gain was significantly more common in younger children (*p* = 0.007), with no other symptoms differing significantly by age group (Supporting Table 1).

**Table 2:** Clinical presentation of children included in the study.

Clinical presentation	General % (*n*) *n* = 34	PPI treatment response % (*n*)		
		Responders *n* = 16	Nonresponders *n* = 18	*p* value
Initial: pretreatment				
Abdominal pain	64.71% (22)	81.25% (13)	50% (9)	0.060^**a**^
Chest pain	29.41% (10)	25% (4)	33.3% (6)	0.440^**a**^
Nausea	26.47% (9)	31.25% (5)	22.22% (4)	0.417^**a**^
Emesis	20.59% (7)	12.5% (2)	27.78% (5)	0.252^**a**^
Regurgitations	61.76% (21)	62.5% (10)	61.11% (11)	0.607^**a**^
Food impaction	47.06% (16)	50% (8)	44.44% (8)	0.508^**a**^
Heartburn	76.47% (26)	93.7% (15)	61.11% (11)	0.030^**a**^^∗^
Dysphagia	32.35% (11)	37.5% (6)	27.78% (5)	0.406^**a**^
Odynophagia	14.71% (5)	18.75% (3)	11.11% (2)	0.441^**a**^
Early satiety	8.82% (3)	12.5% (2)	5.56% (1)	0.455^**a**^
Compensatory measures	20.59% (7)	12.5% (2)	27.78%(5)	0.529^**a**^
Weight loss	14.71% (5)	31.2% (5)	0	0.016^**a**^^∗^
Posttreatment				
Abdominal pain	23.53% (8)	25% (4)	22.22% (4)	0.583^a^
Chest pain	8.82% (3)	12.5% (2)	5.56% (1)	0.455^a^
Nausea	14.71% (5)	12.5% (2)	16.66% (3)	0.559^a^
Emesis	11.76% (4)	12.5% (2)	11.11% (2)	0.652^a^
Regurgitations	23.53% (8)	12.5% (2)	33.3% (6)	0.153^a^
Food impaction	2.94% (1)	0	5.56% (1)	0.529^a^
Heartburn	35.29% (12)	37.5% (6)	33.3% (6)	0.541^a^
Dysphagia	8.82% (3)	0	16.66% (3)	0.136^a^
Odynophagia	0	0	0	NA
Early satiety	2.94% (1)	6.25% (1)	0	0.471^a^
Compensatory measures	5.88% (2)	0	11.11% (2)	0.273^a^

*Note:* The bold values indicate statistically significant values.

^a^Fisher’s exact test.

^∗^Statistical significance at 5%.

### 3.3. Treatment Response

Following initial PPI therapy, 47.1% (16 patients) achieved histological remission and were classified as responders, while 52.9% (18 patients) were nonresponders.

#### 3.3.1. Comparisons Between Responders and Nonresponders

There were no significant differences in sex, age, personal or parental atopic history, and comorbidities between responders and nonresponders (Table 1). Regarding clinical symptoms, heartburn (*p* = 0.030) and weight loss (*p* = 0.016) at baseline were more commonly reported by responders. Both groups experienced a reduction in symptom frequency at follow‐up; however, posttreatment differences were not statistically significant (Table 2).

#### 3.3.2. Histology

Baseline histological parameters did not differ significantly between groups, with mean peak eosinophil counts of approximately 50 eos/hpf in both proximal and distal esophageal biopsies. Basal zone hyperplasia and eosinophil surface layering were also common (Table 3). However, following PPI therapy, responders exhibited significant reductions in peak eosinophil counts in both proximal and distal esophageal biopsies (*p* < 0.005), along with significant improvements in basal zone hyperplasia, eosinophilic abscesses, and eosinophil surface layering (all *p* < 0.005) (Table 3). Notably, the magnitude of eosinophil count reduction (delta between first and second EGD) between baseline and follow‐up was significantly greater among responders compared with nonresponders (*p* < 0.005) (Table 3).

**Table 3:** Histological characteristics of children included in the study.

Histological findings	General % (*n*) *n* = 34	PPI treatment response % (*n*)		
		Responders *n* = 16	Nonresponders *n* = 18	*p* value
Initial: pretreatment				
Mean eos/hpf (IQR)				
(i) Proximal esophagus	50.58 (30–64)	41.43 (22.3–60.5)	58.72 (42.5–74.9)	0.110^**a**^
(ii) Distal esophagus	50.05 (25–70)	48.5 (34.5–62.4)	51.4 (37.3–65.5)	0.778^**a**^
Basal zone hyperplasia	97.06% (33)	93.75% (15)	100% (18)	0.471^**a**^
Eosinophilic abscess	26.47% (9)	18.75% (3)	33.33% (6)	0.285^**a**^
Eosinophilic surface layering	91.18% (31)	93.75% (15)	88.89% (16)	0.545^**a**^
Lamina propria fibrosis	5.88% (2)	0	11.11% (2)	0.487^**a**^
Posttreatment				
Mean eos/hpf (IQR)				
(i) Proximal esophagus	29.32 (0–48)	1.12 (0.19–2.05)	54.3 (32.1–76.6)	**<**0.001^**b**^^∗^
(ii) Distal esophagus	29.52 (1–50)	1.56 (0.31–2.80)	54.3 (34.1–74.6)	**<**0.001^**b**^^∗^
Basal zone hyperplasia	58.82% (20)	12.5% (2)	100% (18)	**<**0.001^**a**^^∗^
Eosinophilic abscesses	17.65% (6)	0	33.33% (6)	0.014^**a**^^∗^
Eosinophilic surface layering	50% (17)	0	94.44% (17)	**<**0.001^**a**^^∗^
Lamina propria fibrosis	0	0	0	NA
Eosinophil count differences between 1st and 2nd EGD				
Proximal delta eos/hpf (IQR)	21.2 (0–44)	40.3 (21.1–59.4)	4.3 (−15.2–23.8)	**<**0.001^**b**^^∗^
Distal delta eos/hpf (IQR)	20.5 (0–48)	46.9 (33.02–60.8)	−2.94 (−19.1–13.2)	**<**0.001^**b**^^∗^

*Note:* The bold values indicate statistically significant values.

Abbreviations: eos/hpf = eosinophil/high‐power field, IQR = interquartile range.

^a^Fisher’s exact test.

^b^Mann–Whitney *U* test.

^∗^Statistical significance at 5%.

#### 3.3.3. Endoscopic Findings

The median interval between baseline and follow‐up endoscopy was 122 days (IQR: 105–149 and range: 55–294 days), with a mean of 136 days. Details of endoscopic findings before and after treatment are available in Supporting Table 2. No statistically significant differences were observed in endoscopic features between responders and nonresponders at either baseline or follow‐up.

## 4. Discussion

This is the first study in Colombia to report histological response to PPI therapy in children with EoE, providing valuable data from a pediatric referral center. We found that 47.1% of studied children achieved histological remission after initial PPI monotherapy, comparable to rates reported internationally [7]. These findings reinforce the role of PPI as an effective first‐line for pediatric EoE, even in regions where epidemiological data are limited.

From a histological standpoint, our analysis went beyond peak eosinophil count reduction, to include other key histopathological markers such as basal zone hyperplasia, eosinophilic abscesses, and eosinophils surface layering, features that reflect chronic epithelial injury, local inflammation, and tissue remodeling. Simultaneous resolution of these parameters in responders suggests that true histological remission in EoE involves a broader resolution of tissue injury, not just meeting an eosinophil count threshold. These findings are consistent with current EoE management guidelines that emphasize histology as the principal criterion for assessing both treatment efficacy and disease progression [2].

Notably, the inclusion of the delta eosinophil count between diagnostic and follow‐up EGD (delta eos/hpf) adds a novel, quantitative dimension to evaluating therapeutic response, supporting the use of dynamic histological parameters as sensitive outcome measures. We found that larger deltas were significantly associated with treatment response, suggesting that this parameter may help identify partial responders who still derive meaningful benefit from therapy.

Importantly, both our results and prior evidence consistently reinforce that histological assessment remains the most critical and reliable marker for diagnosis, evaluation of treatment response, and disease control in EoE. Notably, baseline histological parameters were comparable between groups, indicating a similar degree of esophageal inflammation at the time of diagnosis. This finding aligns with the prior research by Gutiérrez‐Junquera et al., who in a prospective study, showed that baseline histological features did not distinguish responders from nonresponders; although nonresponders exhibited significantly higher mean eosinophil counts, no clear predictive threshold for PPI response could be established [12]. Consequently, no initial histological marker has yet been validated to reliably predict PPI response in pediatric EoE.

Clinically, heartburn, abdominal pain, and regurgitation were the most prevalent baseline symptoms, consistent with the classic EoE phenotype in children. Age‐stratified analysis showed that weight loss or poor weight gain was significantly more frequent in younger patients, underscoring the importance of considering growth compromise as a key presenting symptom of EoE in this age group. However, in older children we did not observe a predominance of symptoms typically associated with fibrotic disease such as abdominal pain, chest pain and food impaction as reported in literature​ [13]. When we analyzed by response groups, heartburn and weight loss at diagnosis were more frequently reported among responders, although this association has been inconsistently observed in the literature [12, 14] potentially reflecting heterogeneity in symptom reporting (from patients and parents report), variability in inflammatory phenotypes or overlap with conditions such as GERD. Notably, both groups demonstrated clinical improvement following therapy, further illustrating the well‐recognized discordance between symptomatic relief and histological remission and reinforcing the necessity of biopsy‐based monitoring in routine EoE management [12].

Endoscopically, no significant differences were identified between responders and nonresponders, either at diagnosis or after treatment. This observation aligns with existing studies using the EREFS score and further supports the notion that endoscopic appearance alone is insufficient to determine disease activity or treatment success [8, 15].

### 4.1. Limitations

This study has several limitations. Its retrospective design and reliance on medical records inherently limit data completeness and standardization and may introduce selection and information bias. The small, single‐center sample size limits the generalizability and statistical power of the findings. No a priori power calculation was performed, and absence of statistical differences may reflect limited power data than true equivalence. Otherwise, due to the sample size, especially after stratification by response status, performing a multivariate analysis to identify independent predictors of PPI response was not feasible.

Symptom data relied on self‐report in older children and parental report in younger children; while practical in routine clinical settings, may be subject to recall bias and reporting variability. Moreover, the absence of use validated, age‐specific symptom scoring tools limits the precision and comparability of symptom assessment across age groups.

It is also important to contextualize some of these results within the framework of the COVID‐19 pandemic, which imposed substantial barriers to clinical care and data collection. Healthcare access restrictions and system disruptions resulted in delays and heterogenous timing of follow‐up endoscopies, as reflected by the wide range observed in procedure intervals. This context may have contributed additional variability in accuracy of outcome assessments, representing an unavoidable limitation of the study period.

To mitigate these limitations, future research should aim for larger sample sizes through multicenter prospective collaborative studies in Colombia and across Latin America. Such efforts would enable greater representation of demographic and phenotypic diversity, facilitate more robust analyses, multivariate models, and adequate statistical power, and improve external validity. Prospective study designs incorporating structured follow‐up protocols, standardized, validated assessment tools for both clinical and histological outcomes, collaborative work and precise protocols of pharmacological criteria selection, and strict following adherence will be essential to favor advancing the understanding of PPI response and to optimize treatment strategies for pediatric EoE.

## 5. Conclusion

This study demonstrates that PPI are effective inducing histological remission in a significant proportion of Colombian children sample with EoE, with results comparable to international literature. Our findings strongly support histological assessment as the cornerstone of follow‐up, since demographic, clinical, and endoscopic variables alone were inadequate to suggest therapeutic response. Nonetheless, the retrospective, single‐center nature and small sample size of this work need cautious interpretation. We propose that larger, prospective, and multicenter investigations will be crucial for identifying reliable predictors of PPI response and advancing individualized care of pediatric EoE in Colombian and Latin American children.

## Conflicts of Interest

The authors declare no conflicts of interest.

## Funding

No funding was received for this manuscript.

## Supporting Information

Supporting Table 1: includes clinical presentation data according to age stratification over and under 10 years, based on mean age.

Supporting Table 2: endoscopic findings pre‐ and posttreatment.

## Supporting information


**Supporting Information** Additional supporting information can be found online in the Supporting Information section.

## Data Availability

Preliminary results from this study were presented as abstracts at two international conferences: the ESPGHAN 56th Annual Meeting 2024 (Milan, Italy; JPGN Supplement Abstract G‐PP089) and the NASPGHAN Annual Meeting 2023 (oral presentation, awarded the Latin America Travel Award). Only the abstracts required by the conference organizers were published in these proceedings. Additionally, the dataset and main results were included in the principal investigator’s master’s thesis in epidemiology, which is available in Spanish in the Los Andes University open repository. This study has not been published as a full article in any peer‐reviewed journal.
