# Supplementary material for: Response to Proton‐Pump Inhibitors Therapy in Pediatric Patients With Eosinophilic Esophagitis in Bogotá, Colombia
Source: Can J Gastroenterol Hepatol. 2025 Nov 25;2025:4633813. doi: 10.1155/cjgh/4633813 (PMC12645149; doi:10.1155/cjgh/4633813)
Supplement: Supplementary file 1 — Supporting Information Additional supporting information can be found online in the Supporting Information section. [file CJGH-2025-4633813-s001.docx]

**SUPPLEMENTARY MATERIAL**

| ***Supplemental Table 1. Clinical presentation by stratified age group*** | **School children**  **(<10 years)**  **n= 14** | **Teenagers (>10 years)**  **n=20** | **p-value** |
| --- | --- | --- | --- |
| **Abdominal pain** | 50% (7) | 75% (15) | 0.128**a** |
| **Chest pain** | 21.43% (3) | 35% (7) | 0.322**a** |
| **Nausea** | 14.29% (2) | 35% (7) | 0.171**a** |
| **Emesis** | 21.43% (3) | 20% (4) | 0.622**a** |
| **Regurgitations** | 71.43% (10) | 55% (11) | 0.272**a** |
| **Food impaction** | 57.14% (8) | 40% (8) | 0.262**a** |
| **Heartburn** | 71.43% (10) | 80% (16) | 0.428**a** |
| **Dysphagia** | 35.71% (5) | 30% (6) | 0.505**a** |
| **Odynophagia** | 21.43% (3) | 10% (2) | 0.328**a** |
| **Early satiety** | 21.43% (3) | 0 | 0.061**a** |
| **Compensatory measures** | 21.43% (3) | 20% (4) | 0.588**a** |
| **Weight loss / poor weight gain** | 35.71% (5) | 0 | **0.007a*** |
| *a. Fisher's exact test*  **Statistical significance at 5%.* | | | |

| ***Supplemental Table 2. Endoscopic characteristics of children included in the study*** | | | | |
| --- | --- | --- | --- | --- |
| **ENDOSCOPIC FINDINGS** | **General % (n)**  **n = 34** | **PPI treatment response % (n)** | | |
|  |  | **Responders**  **n= 16** | **Non-responders n= 18** | ***p-value*** |
| **INITIAL – PRE-TREATMENT:** |  |  |  |  |
| **E**xudates: | 67.65% (23) |  |  |  |
| - Mild grade (<10% surface) | 65.2% (15/23) | 31.25% (5) | 55.56% (10) | 0.151**a** |
| - Severe grade (>10% surface) | 34.7% (8/23) | 18.75% (3) | 27.78% (5) |  |
|  |  |  |  |  |
| **R**ings: | 32.35% (11) |  |  |  |
| - Mild grade | 9.09% (1/11) | 0 | 5.56% (1) |  |
| - Moderate grade | 90.9% (10/11) | 18.75% (3) | 38.89% (7) | 0.196**a** |
| - Severe grade | 0 | 0 | 0 |  |
| **E**dema | 26.47% (9) | 25% (4) | 27.78% (5) | 0.583**a** |
| Vertical **F**urrows | 55.88% (19) | 56.25% (9) | 55.56% (10) | 0.620**a** |
| **S**tricture | 5.88% (2) | 0 | 11.1% (2) | 0.273**a** |
| **POST-TREATMENT:** |  |  |  |  |
|  |  |  |  |  |
| **E**xudates: | 55.88% (19) |  |  |  |
| - Mild grade (<10% surface) | 84.2% (16/19) | 37.5% (6) | 55.56% (10) | 0.060**a** |
| - Severe grade (>10% surface) | 15.8% (3/19) | 0 | 16.67% (3) |  |
| **R**ings: | 26.47% (9) |  |  |  |
| -Mild grade | 22.2% (2/9) | 0 | 11.11% (2) |  |
| -Moderate grade | 77.8% (7/9) | 18.75% (3) | 22.22% (4) | 0.594ª |
| -Severe grade | 0 | 0 | 0 |  |
| **E**dema | 8.82% (3) | 6.25% (1) | 11.11% (2) | 0.545**a** |
| Vertical **F**urrows | 35.29% (12) | 18.75% (3) | 50% (9) | 0.060**a** |
| **S**tricture | 2.94% (1) | 0 | 5.56% (1) | 0.529**a** |
| *a. Fisher's exact test*  **Statistical significance at 5%.* | | | | |
